# Integrating Multi-Omics Data to Identify Novel Disease Genes and Single-Neucleotide Polymorphisms

**DOI:** 10.3389/fgene.2019.01336

**Published:** 2020-01-24

**Authors:** Sheng Zhao, Huijie Jiang, Zong-Hui Liang, Hong Ju

**Affiliations:** ^1^Department of Radiology, The Second Affiliated Hospital of Harbin Medical University, Harbin, China; ^2^Department of Radiology, Jian’an District Centre Hospital of Fudan University, Shanghai, China; ^3^Department of Information Engineering, Heilongjiang Biological Science and Technology Career Academy, Harbin, China

**Keywords:** stroke, genome-wide association study, expression quantitative trait loci, mQTL, SMR, single-nucleotide polymorphisms

## Abstract

Stroke ranks the second leading cause of death among people over the age of 60 in the world. Stroke is widely regarded as a complex disease that is affected by genetic and environmental factors. Evidence from twin and family studies suggests that genetic factors may play an important role in its pathogenesis. Therefore, research on the genetic association of susceptibility genes can help understand the mechanism of stroke. Genome-wide association study (GWAS) has found a large number of stroke-related loci, but their mechanism is unknown. In order to explore the function of single-nucleotide polymorphisms (SNPs) at the molecular level, in this paper, we integrated 8 GWAS datasets with brain expression quantitative trait loci (eQTL) dataset to identify SNPs and genes which are related to four types of stroke (ischemic stroke, large artery stroke, cardioembolic stroke, small vessel stroke). Thirty-eight SNPs which can affect 14 genes expression are found to be associated with stroke. Among these 14 genes, 10 genes expression are associated with ischemic stroke, one gene for large artery stroke, six genes for cardioembolic stroke and eight genes for small vessel stroke. To explore the effects of environmental factors on stroke, we identified methylation susceptibility loci associated with stroke using methylation quantitative trait loci (MQTL). Thirty-one of these 38 SNPs are at greater risk of methylation and can significantly change gene expression level. Overall, the genetic pathogenesis of stroke is explored from locus to gene, gene to gene expression and gene expression to phenotype.

## Introduction

Stroke is a major cerebrovascular disease caused by a transient or permanent decrease of local cerebral blood flow. It is characterized by arterial obstruction (Krishnamurthi et al., 2018), so it is also called cerebral infarction (Dargazanli et al., 2018). According to the World Health Organization, stroke affects more than 15 million people worldwide and directly kills about 5.7 million people. It also causes approximately 5 million people to have a lifelong disability, while about 4.3 million people died due to disability. At present, thrombolytic therapy (Castellanos et al., 2018) (recombinant tissue plasminogen activator) is the only acute treatment for ischemic stroke with a narrow time window (3–4.5 hours). Therefore, only 3.4%–5.2% of patients were treated within the short time window. Researchers have been focusing on how to improve the clinical diagnosis and treatment of cerebral infarction beyond the time window of thrombolysis (Feil et al., 2019).

The occurrence and development of ischemic stroke is affected by a variety of risk factors, such as family history of stroke (Zheng et al., 2019), history of heart disease (Beck et al., 2018), history of diabetes (Zou et al., 2018), history of hypertension, etc. According to the investigation and analysis of Li et al. (2019), the prevalence rate of the family with a family history of stroke is 10.52%. In recent years, a number of genetic association studies have suggested that there are multiple genetic risk factors for ischemic stroke, and multiple risk loci were found to affect the susceptibility to ischemic stroke.

Cacabelos et al. (2018) and Yee et al. (2019) showed that the C7673T polymorphism of APOB gene was significantly associated with the risk of ischemic stroke. Chen et al. (2019), Nordestgaard et al. (2018) confirmed that the polymorphism of ϵ 2,ϵ3,ϵ4 of APOE gene was associated with ischemic stroke. APOB gene and APOE gene are both known ischemic stroke susceptibility genes because of blood lipid level. In addition, many studies have shown that the SG13S114 (rs10507391) polymorphism of ALOX5AP gene and SG13S32 (rs9551963) polymorphism are associated with susceptibility to ischemic stroke. Zheng et al., (2018) found that carriers of SG13S114 polymorphism TT/TA genotype of ALOX5AP gene had a higher risk of acute cerebral infarction. Naderi et al. (2019) showed that SG13S114 polymorphism of ALOX5AP gene was associated with acute cerebral infarction. Previous genetic studies have found that some ischemic stroke susceptibility genes on chromosome 14, such as GCH1 gene (Wei et al., 2018), MEG3 gene (Han et al., 2018), MMP-14 gene (Elgebaly et al., 2019), PRKCH gene (Krupinski et al., 2018), are associated with the risk of ischemic stroke.

Genome-wide association study (GWAS) reveals candidate loci, susceptible genes and their loci related to the occurrence, development and treatment of diseases by genome-wide high-density genetic markers (Pei Li and Wang, 2015; Cheng et al., 2019a; Cheng et al., 2019b). Since 2009, GWAS has been widely used to explore and excavate candidate gene loci related to new types of stroke. GWAS is generally believed to be able to identify some previously undetected or identified biological markers related to stroke (Ye et al., 2018; Cheng et al., 2019c), and because of its large sample size, it can minimize false positive results. The National Institute of Neurological Diseases (NIND) has conducted the largest and most comprehensive GWAS to explore the genetic loci of stroke and its subtypes. The results supported the previously established genetic association of ischemic stroke. New loci on chromosome 1p13 (such as rs12122341 of TSPAN2 gene) have been found to be associated with ischemic stroke. Although GWAS has many advantages and is widely used, it is still very hard to understand the role of nucleotide polymorphism (SNP) loci in diseases from the huge results of GWAS.

Therefore, recently many researchers have tried to integrate GWAS with expression quantitative trait loci (eQTL) to mine the disease-related genes (Cheng et al., 2018a; Cheng et al., 2018b). Since eQTL conveys gene expression information and GWAS conveys disease-related SNPs information, combining the two datasets, we could know the loci which are associated with diseases because of affecting other genes expression. Zhao et al. (2019) found many Alzheimer’s disease-related genes and SNPs by GWAS and eQTL. Asthma-related genes were identified by Li et al. (2015). by integrating GWAS and eQTL. Systematic integration of Brain eQTL and GWAS were done by Luo et al. (2015) and they identified ZNF323 as a novel Schizophrenia risk gene.

Zhu et al. (2016) generalized Mendelian randomization to SMR. SMR is used to test the association between a trait and the expression level of each gene across the whole genome using summary data from GWAS and eQTL studies. SMR is a common tool to identify the genes whose expression levels are associated with a complex trait because of pleiotropy. Twenty-eight GWAS datasets are used by Pavlides et al. (2016) to find genes whose expression levels were associated with complex phenotype. Bone mineral density (BMD)-related genes are studied by Meng et al. (2018) using SMR. SMR is also used to identify genes and pathways for Amyotrophic Lateral Sclerosis by Du et al. (2017). Fan et al. (2017) found 6 genes are associated with neuroticism by SMR. Liu et al. (2018) used SMR on doing research on Obesity and found 20 BMI associated genes. Veturi and Ritchie (2018) compared two popular methods: MP and SMR by different datasets. Though these scholars’ researches, we could judge that SMR is an effective tool. In this paper, summary-level data mendelian randomization (SMR) is used to integrate GWAS and eQTL datasets. In this way, the most functionally relevant genes at the loci identified in GWAS for stroke are found.

## Methods

### Work Frame

**Figure 1:**
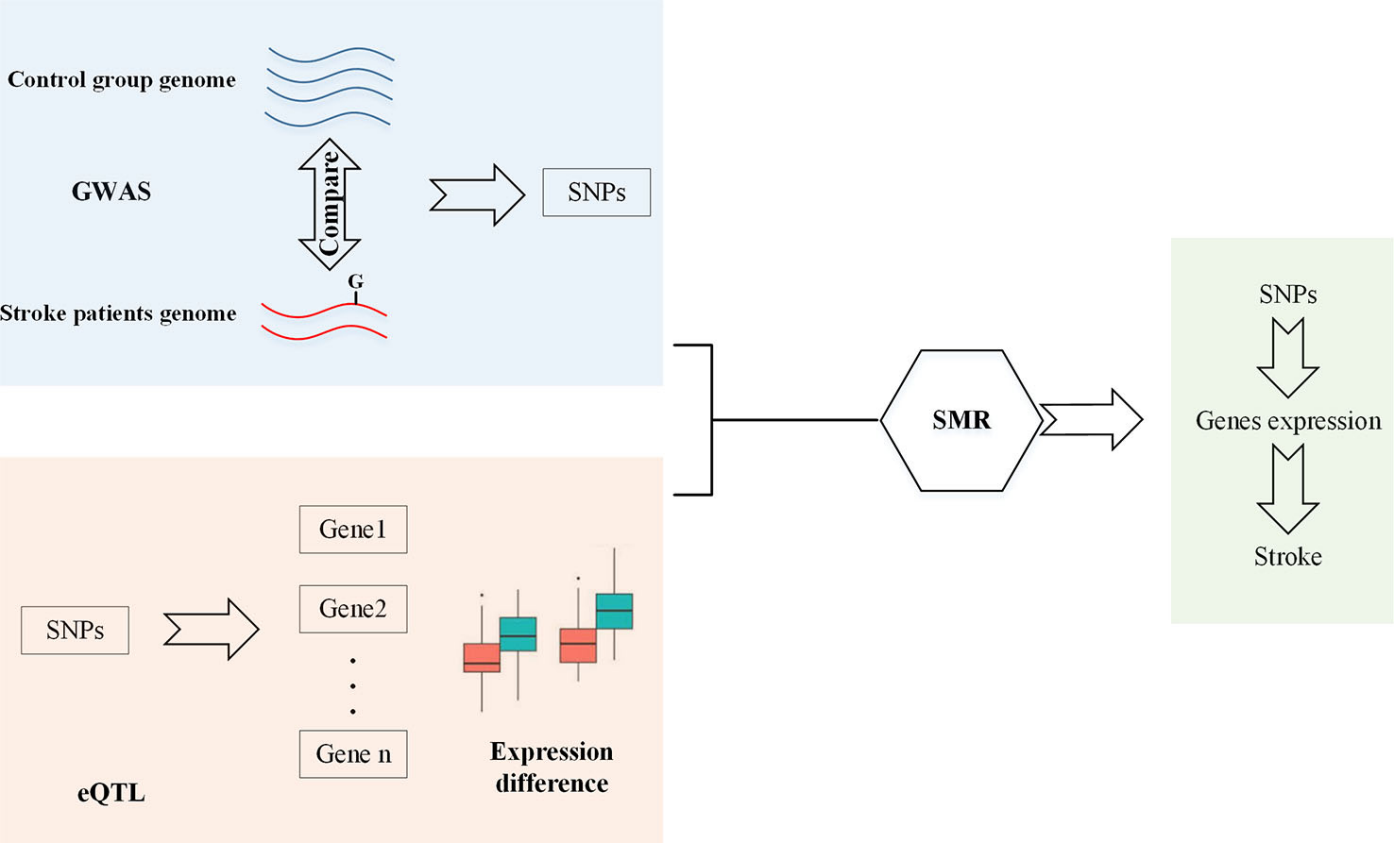
Workflow of SMR.

As shown in **Figure 1**, since GWAS has identified SNPs which are related to stroke, and eQTL has identified SNPs which can affect genes expression, SMR is used to identify SNPs that can change gene expression and this should be the reason that they are associated with stroke. Therefore, firstly, we should obtain GWAS and eQTL data. Then, we checked the overlap between these two datasets. Finally, SMR is used to screen SNPs.

### SMR

z in summary data level Mendelian Randomization (SMR) is a genetic variant (SNP), x is the expression level of a gene and y denotes the trait, then the two-step least-squares estimate of the effect of x on y from an MR analysis is:

(1) $${\hat{b}}_{xy}={\hat{b}}_{zy}/{\hat{b}}_{zx}$$

${\hat{b}}_{zy}$ and ${\hat{b}}_{zx}$ are the least-squares estimates of y and x on z, respectively. Then, ${\hat{b}}_{xy}$ denotes the effect size of x on y without confounding from non-genetic factors. The variance of ${\hat{b}}_{xy}$ is:

(2) $$T_{MR}={\hat{b}}_{xy}^{2}/\mathrm{var}({\hat{b}}_{xy})$$

Here, *T_MR_* obeys a chi-square distribution with a degree of freedom of 1. As we can see in equation (Dargazanli et al., 2018), MR requires genotype, gene expression and phenotype to be measured on the same sample. However, Zhu et al. have proved that the power of detecting ${\hat{b}}_{xy}$ can be greatly increased using a two-sample MR analysis. Therefore, the *T_MR_* can be replaced by *T_SMR_*.

(3) $$T_{SMR}={\hat{b}}_{xy}^{2}/\mathrm{var}({\hat{b}}_{xy})\approx \frac{z_{zy}^{2}z_{zx}^{2}}{z_{zy}^{2}+z_{zx}^{2}}$$

*z_zy_* is the z statistics from GWAS and *z_zx_* is the z statistics from eQTL.

## Results

### Data Description

#### GWAS

We used the data from Malik et al.’s research. Eight GWAS datasets are used. **Table 1** shows the detailed information about these data.

**Table 1:** GWAS data description.

Dataset	Disease	Sample
GWAS 1	ischemic stroke	Europeans (40,585 cases; 406,111 controls)
GWAS 2	ischemic stroke	trans-ethnic meta-analysis (67,162 cases; 454,450 controls)
GWAS 3	large artery stroke	Europeans (40,585 cases; 406,111 controls)
GWAS 4	large artery stroke	trans-ethnic meta-analysis (67,162 cases; 454,450 controls)
GWAS 5	cardioembolic stroke	Europeans (40,585 cases; 406,111 controls)
GWAS 6	cardioembolic stroke	trans-ethnic meta-analysis (67,162 cases; 454,450 controls)
GWAS 7	small vessel stroke	Europeans (40,585 cases; 406,111 controls)
GWAS 8	small vessel stroke	trans-ethnic meta-analysis (67,162 cases; 454,450 controls)

We collected GWAS data for four different types of stroke (ischemic stroke, large artery stroke, cardioembolic stroke, small vessel stroke).

**Figure 2:**
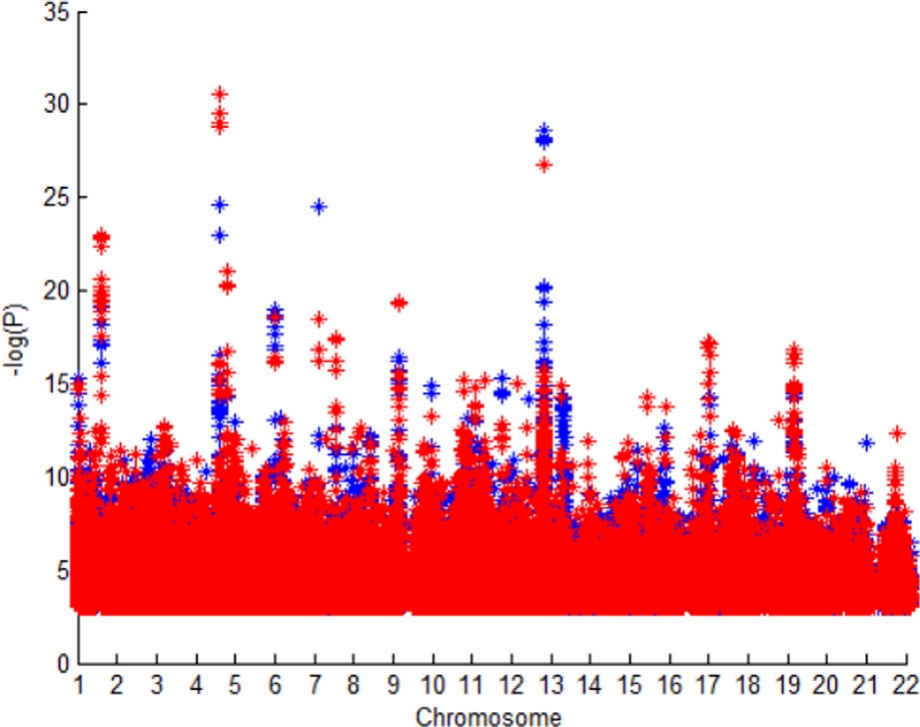
P value of SNPs in GWAS1 and GWAS2.

**Figure 2** shows P value of SNPs in GWAS1 and GWAS2. The SNPs are almost same in these GWAS dataset, but difference races cause the difference of P value. We could know different races have different stroke susceptibility genes.

#### eQTL

eQTL data is from a meta-analysis of GTEx brain (Consortium G, 2017), CMC (Fromer et al., 2016), and ROSMAP (Ng et al., 2017). All the data are from brain. Only SNPs within 1Mb distance from each probe are available. The estimated effective n is 1,194.

#### mQTL

mQTL used in this paper is a set of brain data from a meta-analysis of ROSMAP (Ng et al., 2017), Hannon et al. (2016) and Jaffe et al. (2016). In the ROSMAP data, only SNPs within 5Kb of each DNA methylation probe are available. In the Hannon et al. data, only SNPs within 500Kb distance from each probe and with PmQTL < 1.0e-10 are available. In the Jaffe et al. data, only SNPs within 20Kb distance from each probe and with FDR < 0.1 are available. The estimated effective n is 1,160.

### Four Kinds of Stroke

Ischemic stroke is a kind of stroke which caused by arterial obstruction. It accounts for approximately 85% of the total. large artery stroke and cardioembolic stroke are the subgroup of this kind of this stroke.

Large artery stroke is caused by blood clots (thrombus) which are formed in the neck or cerebral arteries. There may be accumulation of fatty deposits (often referred to as plaques) in these arteries.

Cardioembolic stroke is caused by blood clots that reach the brain and blocks the blood vessels. A common cause is the formation of blood clots in the two upper atrial rhythm abnormalities of the heart (atrial fibrillation).

Small vessel stroke is actually a transient stroke symptom that usually lasts only a few minutes. small vessel stroke is caused by transient blood supply to specific parts of the brain and does not cause significant persistent effects on patients. However, it is generally believed that the risk of stroke after small vessel stroke is higher.

### SNPs and Genes for Ischemic Stroke

10 SNPs which change six genes expression are screened by Europeans dataset and 11 SNPs which change five genes expression are screened by trans-ethnic dataset.

As we can see in **Table 2**, HSD17B12 is overlapped in the two tests. Moreno et al. (2018) found upregulation of HSD17B12 is associated ischemic stroke using 82 cases and 67 controls. ALDH2 is generally considered as a gene (Guo et al., 2013) which can protect against ischemic stroke, because overexpression of ALDH2 rescued neuronal survival against 4-HNE treatment in PC12 cells (Lee et al., 2012). These two genes show the accuracy of our results.

**Table 2:** SMR results of ischemic stroke.

SNP	P-value	Gene
Europeans dataset		
rs9651613	4.17E-06	HSD17B12
rs648997	5.72E-06	ALDH2
rs11065976	6.36E-06	ALDH2
rs4286007	6.70E-06	CKAP2
rs847892	7.79E-06	ALDH2
rs66480035	7.97E-06	ALDH2
rs532436	7.99E-06	SURF1
rs487399	8.21E-06	CEP192
rs11618716	8.80E-06	CKAP2
rs11620062	9.24E-06	CKAP2
Trans-ethnic dataset		
rs9651613	3.58E-07	HSD17B12
rs10838185	5.14E-06	HSD17B12
rs6599175	5.42E-06	ULK4
rs6801343	5.55E-06	ULK4
rs9874975	5.70E-06	ULK4
rs12774577	7.99E-06	C10orf32
rs10400343	8.38E-06	HSD17B12
rs3087681	8.47E-06	C10orf32
rs2371623	8.81E-06	ULK4
rs9825741	9.00E-06	ULK4
rs11191606	9.04E-06	C10orf32

### SNPs and Genes for Large Artery Stroke

None SNP is screened by Europeans dataset for large artery stroke. Three SNPs which correspond one gene ‘C3orf18’ are screened by trans-ethnic dataset.

Phenotypes for C3orf18 Gene include Decreased homologous recombination repair frequency, Decreased ionizing radiation sensitivity, Upregulation of Wnt pathway, Increased vaccinia virus (VACV) infection, Mildly decreased CFP-tsO45G cell surface transport. It is considered to be associated with cognitive function measurement.

### SNPs and Genes for Cardioembolic Stroke

11 SNPs are significant in Europeans dataset and trans-ethnic dataset. rs3807989 is screened more than one time in Europeans dataset because it can affect more than one gene expression. Both CAV1 and CAV2’s expression can be changed by this SNP.

As we can see in **Table 3**, 6 genes and 3 genes are screened by SMR in Europeans dataset and Trans-ethnic dataset, respectively. Three of them are overlapped.

**Table 3:** SMR results of cardioembolic stroke.

SNP	P-value	Gene
Europeans dataset		
rs3807989	2.03E-05	CAV1
rs532436	4.03E-05	SURF1
rs72790984	4.68E-05	PLEKHH2
rs11773845	4.96E-05	CAV1
rs4745721	4.96E-05	ECD
rs1997571	5.62E-05	CAV1
rs507666	6.08E-05	SURF1
rs1997572	6.20E-05	CAV1
rs9313620	6.27E-05	BNIP1
rs76192127	6.35E-05	ECD
rs3807989	6.58E-05	CAV2
rs2519093	7.40E-05	SURF1
rs600038	9.67E-05	SURF1
Trans-ethnic dataset		
rs4745721	2.21E-05	ECD
rs76192127	2.87E-05	ECD
rs532436	3.37E-05	SURF1
rs507666	4.15E-05	SURF1
rs616154	5.26E-05	SURF1
rs72790984	5.60E-05	PLEKHH2
rs2519093	5.92E-05	SURF1
rs72790983	6.37E-05	PLEKHH2
rs559723	7.26E-05	SURF1
rs183153921	7.34E-05	ECD
rs3878005	9.62E-05	ECD

### SNPs and Genes for Small Vessel Stroke

13 SNPs and 4 SNPs are significant in Europeans dataset and trans-ethnic dataset, respectively. None of these SNPs or their corresponding genes are overlapped in these two tests. As we can see in **Table 4**, although no overlap is found between these two tests, some genes are overlapped between cardioembolic stroke and small vessel stroke.

**Table 4:** SMR results of small vessel stroke.

SNP	P-value	Gene
Europeans dataset		
rs3807989	2.03E-05	CAV1
rs532436	4.03E-05	SURF1
rs72790984	4.68E-05	PLEKHH2
rs11773845	4.96E-05	CAV1
rs4745721	4.96E-05	ECD
rs1997571	5.62E-05	CAV1
rs507666	6.08E-05	SURF1
rs1997572	6.20E-05	CAV1
rs9313620	6.27E-05	BNIP1
rs76192127	6.35E-05	ECD
rs3807989	6.58E-05	CAV2
rs2519093	7.40E-05	SURF1
rs600038	9.67E-05	SURF1
Trans-ethnic dataset		
rs2501966	3.53E-06	CENPQ
rs6599175	4.49E-06	ULK4
rs2501965	4.77E-06	CENPQ
rs9874975	6.07E-06	ULK4

### SNPs Changes Gene Expression Level by Methylation

Since both genetic and environmental factors are key to cause stroke, while methylation plays an important role in the interaction between environmental factors and genetic expression, we assumed that some of the SNPs identified above are at greater risk of methylation and can change gene expression levels.

**Figure 3:**
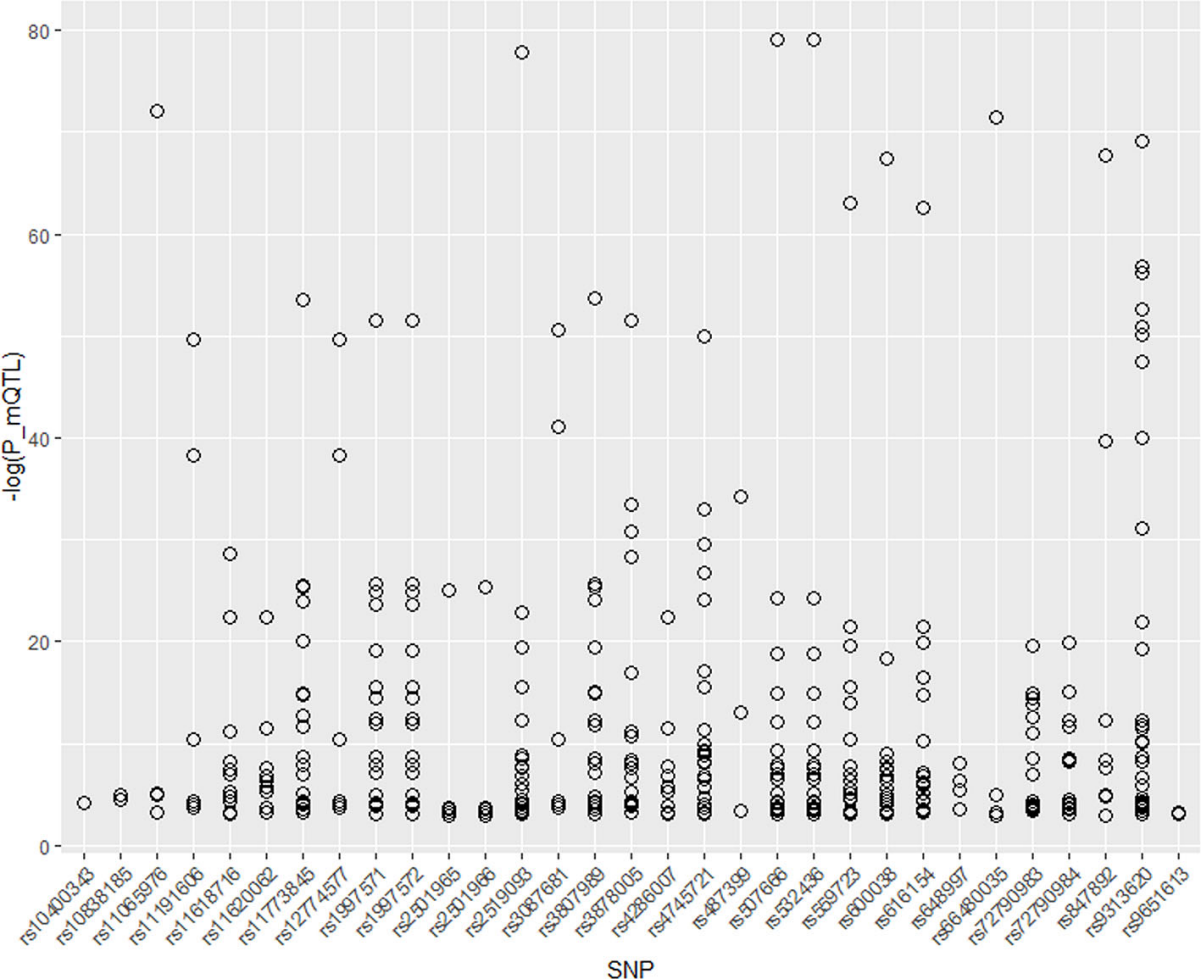
P value of 31 significant SNPs in mQTL.

Therefore, we integrated the SNPs found above with mQTL data for research. Thirty-eight unique SNPs are found in four different types of stroke. Thirty-one of these 38 SNPs are significant in mQTL dataset. We draw the P value of these 31 SNPs as **Figure 2**. As shown in **Figure 3**, most of these SNPs are associated with several genes expression. In addition, most of SNPs have a quite low P value, which means that they can significant change the expression of genes.

### Case Study

#### ULK4

Guo et al. (2016) have found that genetic variants in LRP1 and ULK4 are associated with acute aortic dissections. In their paper, they also mentioned that ULK4 may contribute stroke.

#### CAV1

Shyu et al. (2017) discussed association of eNOS and CAV1 gene polymorphisms with susceptibility risk of large artery atherosclerotic stroke. A tendency toward an increased LAA stroke risk was significant in carriers with the eNOS Glu298Asp variant in conjunction with the G14713 A and T29107A polymorphisms of the CAV1 (aOR = 2.03, P-trend = 0.002).

#### CAV2

Jolobe (2012) found that recurrent stroke is because of a novel voltage sensor mutation in CAV2. They compared stroke mouse and normal mouse to obtain this conclusion.

## Conclusions

Stroke is the primary cause of disability in adults, which constitutes a serious public health burden. Stroke is generally believed to be caused by genetic and environmental factors. Therefore, in this paper, we identified stroke-related genes and loci from both genetic and environmental aspects.

GWAS identified a large number of stroke-related SNPs, which were difficult to explain. We tried to identify the pathogenesis of significant SNPs by combining SMR with eQTL data. Since eQTL shows the SNPs that can significantly change genes expression and GWAS shows the SNPs that are significant related to stroke, we combined these two data to identify the genes whose expression levels are associated with stroke because of pleiotropy.

38 SNPs which cause changes in 14 genes expression were found by 8 GWAS data and brain eQTL. Those 8 GWAS data are from two different races sample and include four types of stroke (ischemic stroke, large artery stroke, cardioembolic stroke, small vessel stroke). CAV1, SURF1, PLEKHH2, ECD, BNIP1, CAV2 are found to be associated with cardioembolic stroke and Small vessel stroke in Europeans. ULK4 is a susceptibility gene for ischemic stroke and small vessel stroke.

Since methylation (Lv et al., 2019) plays an important role in the interaction between environmental factors and genetic expression, we tried to find out whether 38 SNPs are affected by methylation and lead to the changes in other genes expression levels. Thirty-one of these 38 SNPs are significant in mQTL data and most of them can affect more than one gene expression.

Overall, integrating GWAS with eQTL, we found 38 SNPs and 14 genes are related to stroke by SMR. Thirty-one of 38 SNPs are at high risk of methylation which can also cause changes in gene expression. These findings serve as a guide to understanding the pathogenesis of stroke at the molecular level.

## Data Availability Statement

All the datasets used in this paper could be downloaded from GWAS: ftp://ftp.ebi.ac.uk/pub/databases/gwas/summary_statistics/MalikR_29531354_GCST006908/MEGASTROKE.2.AIS.EU

R.out ftp://ftp.ebi.ac.uk/pub/databases/gwas/summary_statistics/MalikR_29531354_GCST005843/MEGASTROKE.2.AIS.TR

ANS.out ftp://ftp.ebi.ac.uk/pub/databases/gwas/summary_statistics/MalikR_29531354_GCST006907/MEGASTROKE.3.LAS.EU

R.out ftp://ftp.ebi.ac.uk/pub/databases/gwas/summary_statistics/MalikR_29531354_GCST005840/MEGASTROKE.3.LAS.TR

ANS.out ftp://ftp.ebi.ac.uk/pub/databases/gwas/summary_statistics/MalikR_29531354_GCST005842/MEGASTROKE.4.CES.TR

ANS.out ftp://ftp.ebi.ac.uk/pub/databases/gwas/summary_statistics/MalikR_29531354_GCST006910/MEGASTROKE.4.CES.EU

R.out ftp://ftp.ebi.ac.uk/pub/databases/gwas/summary_statistics/MalikR_29531354_GCST005841/MEGASTROKE.5.SVS.TR

ANS.out ftp://ftp.ebi.ac.uk/pub/databases/gwas/summary_statistics/MalikR_29531354_GCST006909/MEGASTROKE.5.SVS.EU

R.out eQTL: https://cnsgenomics.com/software/smr/#eQTLsummarydata

mQTL: https://cnsgenomics.com/software/smr/#mQTLsummarydata.

## Author Contributions

HuJ, Z-HL, and HoJ conceived and designed the experiments. SZ analyzed data. SZ, HuJ, Z-HL, and HoJ wrote this manuscript. All authors read and approved the final manuscript.

## Funding

This study was supported by grants from the National Natural Science Foundation of China (81671760 and 81873910), Scientific Research Transformation Special Fund of Heilongjiang Academy of Medical Sciences (2018415);Scientific Research Project of Health and Family Planning Commission of Heilongjiang Province (201812 and 201622), National Natural Science Foundation of China (81871423), and Shanghai Municipal Commission of Health and Family Planning (20160064).

## Conflict of Interest

The authors declare that the research was conducted in the absence of any commercial or financial relationships that could be construed as a potential conflict of interest.
